# Gender disparities in Behçet’s syndrome: identifying distinct phenotypes through cluster analysis

**DOI:** 10.1007/s12026-024-09498-1

**Published:** 2024-05-29

**Authors:** Gamze Kılıç, Kemal Faruk Körüklü, Muhammed Galip Kumcu, Elif Çakır, Murat Karkucak, Erkan Kılıç

**Affiliations:** 1 Division of Rheumatology, Department of PMR, Karadeniz Technical University School of Medicine, Trabzon, Turkey; 2 Rheumatology Clinic, Kanuni Training and Research Hospital, Trabzon, Turkey

**Keywords:** Behçet’s syndrome, Gender, Clinical phenotype, Cluster

## Abstract

Behçet’s syndrome (BS) is a complex, multi-systemic disorder with a global occurrence, notably concentrated along the Silk Road. This study aimed to investigate gender-specific expressions and clinical phenotypes in BS patients within the Eastern Black Sea Region of Turkey. A total of 290 BS patients were retrospectively analyzed between January 2013 and December 2023. Demographic characteristics, clinical manifestations, medical treatment, and pathergy test results were obtained from a review of medical records. The mean age was 45.79 ± 13.05, with a male-to-female ratio of 48.6:51.4. Male patients had higher papulopustular lesions (*p* < 0.001) and ocular involvement (*p* = 0.036), while females showed more frequent genital ulcers (*p* = 0.032). Medication usage showed gender-based variations, notably higher corticosteroid, azathioprine, and tumor necrosis factor-alpha inhibitor (TNFi) use in males (*p* < 0.001). Cluster analysis revealed five distinct clusters, each with unique features and gender predominance. Cardiovascular type, ocular type, and skin type predominantly featured male patients, while joint involvement type and neurologic and mucosal involvement type were more prevalent among female patients with BS. This research contributes valuable insights into the gender-related clinical variations of BS within a specific geographic region, fostering a more comprehensive understanding of this challenging syndrome. The identification of distinct clinical phenotypes facilitates the development of tailored treatment strategies, potentially leading to improved outcomes for patients with BS.

## Introduction

Behçet’s syndrome (BS) is a complex and multi-systemic disorder characterized by systemic vasculitis affecting numerous organs and systems. It commonly presents in individuals during early adulthood [1]. The exact cause of BS remains unknown, but it is believed to involve a combination of genetic, environmental, and immunological factors [2]. The disease has a heterogeneous range of clinical phenotypes including mucocutaneous, articular, ocular, vascular, neurological, and gastrointestinal manifestations [3]. Although oral ulceration is frequently the initial manifestation of BS, patients may exhibit varying combinations of clinical features over time.

Despite BS having a global occurrence, it is notably more prevalent in populations with historical roots along the Silk Road, encompassing regions the Mediterranean, the Middle East, and East Asia [4]. Moreover, the frequency of clinical manifestations, as well as the severity of BS, varies among different geographical regions [5–7]. While neurological manifestations are more commonly seen in Caucasians, cardiac manifestations occur more frequently in Asian and Middle Eastern patients compared to Caucasians [5].

Behçet’s syndrome is generally accepted as a condition with a male predominance, but male-to-female ratio may vary between endemic and non-endemic areas [4, 8, 9]. To date, several studies exist regarding gender differences in BS. A comprehensive study involving BS patients from diverse populations revealed significant gender-specific variations, with males exhibiting a notably higher genetic risk score, especially in the human leukocyte antigen (HLA) class I region, compared to female patients [10]. Additionally, observational studies in different geographic regions have underscored gender-associated clinical differences in BS [6, 11–15]. In contrast, some studies from different geographic regions did not find any gender differences in BS [7]. Recognizing gender-specific patterns in BS is crucial for early diagnosis and tailoring effective treatment strategies and interventions to individualized needs. Therefore, the primary aim of this study was to investigate differences in clinical manifestations of BS according to gender within the Eastern Black Sea Region of Turkey, and the secondary aim was to assess the cluster phenotype of patients with BS.

## Material and method

It was designed as a single-center, retrospective clinical study. Between January 2013 and December 2023, a total of 290 BS patients were included from our outpatient clinic of rheumatology. This single-center, retrospective clinical study, utilizing data collected between January 2013 and December 2023, included 290 BS patients from our outpatient rheumatology clinic. The inclusion criteria involved adult patients with BS according to the classification criteria of the International Study Group for Behçet’s Disease [16]. The study protocol was approved by the local ethics committee (20.07.23/24237859-459).

Demographic characteristics, current medical treatment and the results of pathergy test were obtained from a review of medical records. The assessment of clinical manifestations related to BS during the follow-up included a comprehensive review of the patients’ symptoms, physical examinations, laboratory analyses, and imaging studies. Ocular involvement in BS, such as uveitis or retinal vasculitis, was considered after being diagnosed by an ophthalmologist. Neurological involvement was taken into consideration after ruling out other potential causes. Vascular involvement, which included venous and arterial thrombosis as well as arterial aneurysm, was considered based on both clinical and imaging results. Additionally, other clinical manifestations of BS, including mucocutaneous and articular manifestations, were also documented.

### Statistical analysis

The statistical analysis utilized the SPSS software package (version 23.0, IBM Corporation, Armonk, NY, USA). To assess the normality of data distribution, the Kolmogorov-Smirnov test was employed. Descriptive statistics summarized the demographic and clinical characteristics of the patients, with categorical variables reported as *n* (%), and continuous data expressed as mean ± standard deviation (SD). Group differences were assessed using Pearson’s Chi-square for categorical variables and *t*-test for continuous variables. In the domain of cluster analysis, a sophisticated statistical approach for identifying patterns or groups within a dataset, the two-step method was applied. This involved an initial hierarchical clustering process followed by a k-means clustering step, facilitating a robust and comprehensive examination of data patterns. A significance level of *p* < 0.05 was applied to determine statistical significance across all analyses.

## Results

A total of 290 patients with BS, with a mean age of 45.79 ± 13.05, were included in the study. Of the patients, 149 (51.4%) were female and 141 (48.6%) were male. The similarities and differences between female and male patients are presented in Table 1.

**Table 1:** Demographic and clinical finding categorized by gender

	Female (*n* = 149)	Male (*n* = 141)	*p*
Age, year	46.98 ± 13.27	44.52 ± 12.75	0.109
Age at diagnosis (*n* = 171)	32.35 ± 11.26	31.02 ± 10.70	0.432
Pathergy (*n* = 219)	23 (21.3)	32 (28.8)	0.199
Drug usage (*n* = 236)			
Corticosteroids	29 (25.4)	57 (46.7)	0.001
NSAID	5 (4.4)	7 (5.1)	0.637
Colchicum	103 (90.4)	103 (84.4)	0.172
TNFi	8 (7.0)	19 (15.6)	0.039
Azathioprine	29 (25.4)	61 (50.0)	< 0.001
Methotrexate	2 (1.8)	0	0.232
Cyclosporin	0	4 (3.3)	0.123
Cyclophosphamide	0	2 (1.6)	0.498
CRP (*n* = 226)	7.06 ± 23.27	9.93 ± 21.01	0.332
ESR (*n* = 211)	17.38 ± 14.58	11.83 ± 12.06	0.003

*NSAID* non-steroidal anti-inflammatory drug, *TNFi* tumor necrosis factor inhibitor, *CRP* C-reactive protein, *ESR* erythrocyte sedimentation rate

The mean ages of females and males were 46.98 ± 13.27 and 44.52 ± 12.75, respectively (*p* = 0.109). The age at diagnosis was similar between genders, with females at 32.35 ± 11.26 and males at 31.02 ± 10.70 (*p* = 0.432). Pathergy was observed in 21.3% of females and 28.8% of males, with no significant difference found (*p* = 0.199).

Analysis of drug usage indicated notable gender-based variations. Corticosteroid usage was higher in males (46.7%) compared to females (25.4%) with a significant *p*-value of 0.001. Tumor necrosis factor-alpha inhibitor (TNFi) usage was more prevalent in males (15.6%) compared to females (7.0%), showing a significant difference (*p* = 0.039). Azathioprine usage was significantly higher in males (50.0%) compared to females (25.4%, *p* < 0.001). Other drug usages, including nonsteroidal anti-inflammatory drugs (NSAIDs), colchicum, methotrexate, cyclosporin, and cyclophosphamide, did not exhibit significant gender-based differences.

**Fig. 1:**
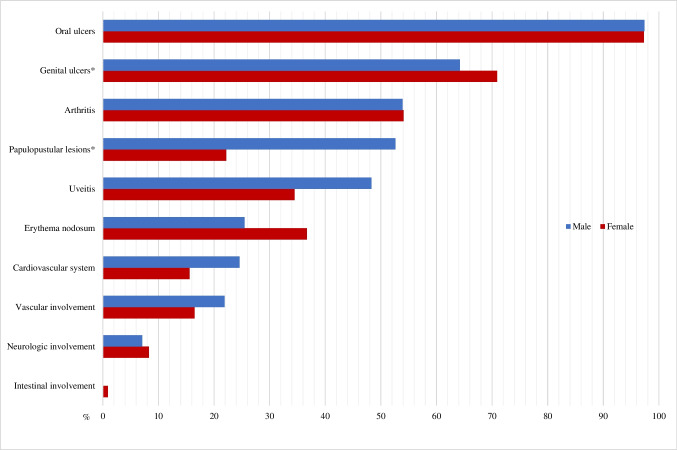
Clinical features of Behçet’s syndrome in male and female groups

Clinical manifestations are summarized in Fig. 1, revealing distinct gender variations in the prevalence of certain conditions and providing valuable insights into gender-specific aspects. Oral manifestations exhibited high prevalence in both females (97.3%) and males (97.4%), with no statistically significant difference. Genital ulcers showed a notable difference in occurrence, with 70.9% in females compared to 64.2% in males (*p* = 0.032). For erythema nodosum (EN), females displayed a higher percentage (36.7%) compared to males (25.5%), though the difference did not reach statistical significance (*p* = 0.072). Papulopustular eruption (PPE) demonstrated a substantial gender disparity, with a prevalence of 22.2% in females and a significantly higher 52.6% in males (*p* < 0.001). Arthritis had a comparable prevalence in both females (54.1%) and males (53.9%), with no significant difference noted (*p* = 0.974). Ocular manifestations showed a higher prevalence male (48.3%), compared to females (34.5%), and the difference was statistically significant (*p* = 0.036). Cardiovascular manifestations had a prevalence of 15.6% in females and 24.6% in males, with a borderline *p*-value of 0.095. Peripheral vascular manifestations exhibited no significant gender-based difference, with a prevalence of 16.5% in females and 21.9% in males (*p* = 0.305). Neurologic manifestations demonstrated a low occurrence in both females (8.3%) and males (7.1%), with no significant difference observed (*p* = 0.756). Gastrointestinal (GIS) manifestation was observed in only one female.

### Cluster analysis

Five clusters were generated with distinct features. The characteristics of each cluster were listed in Table 2. Due to missing data, 212 patients were included in the cluster analysis, and only one patient with GIS involvement was excluded from the cluster analysis.

**Table 2:** Comparison of clusters for clinical findings

	Cluster 1 *n* = 36 (17.0%)	Cluster 2 *n* = 19 (9.0%)	Cluster 3 *n* = 75 (35.4%)	Cluster 4 *n* = 46 (21.7%)	Cluster 5 *n* = 36 (17.0%)
Age	45.42 ± 11.66	47.37 ± 15.72	44.20 ± 13.62	45.85 ± 9.83	45.89 ± 14.04
Diagnosis age	31.79 ± 11.75	36.73 ± 13.84	30.81 ± 9.76	32.93 ± 11.21	30.30 ± 10.65
Male/female	21/15	10/9	43/32	21/25	12/24
Oral ulcer	34 (94.4)	15 (78.4)	75 (100%)	46 (100)	36 (100)
Genital ulcer	24 (66.7)	0	44 (58.7)	30 (65.2)	36 (100)
Erythema nodosum	17 (47.2)	0	46 (61.3)	0	0
Papulopustular lesion	15 (41.7)	0	50 (66.7)	13 (28.3)	0
Arthritis/joint	19 (52.8)	10 (52.6)	33 (44)	46 (100)	2 (5.6)
Ocular	16 (44.4)	19 (100)	32 (42.7)	3 (6.5)	14 (38.9)
Cardiovascular	36 (100)	1 (5.3)	2 (2.7)	0	0
Vascular	36 (100)	1 (5.3)	0	0	1 (2.8)
Neurologic	0	3 (15.8)	5(6.7)	0	6 (16.7)

#### Cluster 1 (*n* = 36, 17.0%): cardiovascular type, (male-to-female ratio 1.4:1)

Cluster 1, comprising 36 individuals, demonstrates a moderate prevalence within the study population. The average age of this cluster is 45.42 ± 11.66, with an onset of symptoms occurring at 31.79 ± 11.75 years. The male-to-female ratio in Cluster 1 is 1.4:1. All patients had cardiac and vascular involvement. Noteworthy findings include a high occurrence of oral ulcers (94.4%) and a considerable presence of genital ulcers (66.7%) and joint manifestations (52.8%).

#### Cluster 2 (*n* = 19, 9.0%): ocular type, late-onset, (male-to-female ratio, 1.11:1)

Cluster 2 consists of 19 individuals, with an average age of 47.37 ± 15.72 higher than other clusters. The onset of symptoms occurs at 36.73 ± 13.84 years. The male-to-female ratio in Cluster 2 is 1.11:1. Notably, this cluster is characterized by a high prevalence of ocular manifestations (100%) and a relatively lower occurrence of oral ulcers (78.4%) and arthritis (52.6%).

#### Cluster 3 (*n* = 75, 35.4%): skin type, (male-to-female ratio, 1.34:1)

Cluster 3, the largest cluster with 75 individuals, exhibits a slightly younger average age of 44.20 ± 13.62 and an onset of symptoms at 30.81 ± 9.76 years. The male-to-female ratio is 1.34:1. Notable features include a universal presence of oral ulcers (100%) and a substantial prevalence of both genital ulcers (58.7%) and highest EN (61.3%) and papulopustular lesions (66.7).

#### Cluster 4 (*n* = 46, 21.7%): joint involvement type, (male-to-female ratio, 0.84:1)

Cluster 4, encompassing 46 individuals, demonstrates an average age of 45.85 ± 9.83, with an onset of symptoms at 32.93 ± 11.21 years. The male-to-female ratio in Cluster 4 is 0.84:1. This cluster stands out for its high prevalence of arthritis/joint manifestations (100%), oral ulcers (100%), and a substantial presence of genital ulcers (65.2%).

#### Cluster 5 (*n* = 36, 17.0%): neurologic and mucosal involvement type, (male-to-female ratio, 0.5:1)

Cluster 5, composed of 36 individuals, displays average age of 45.89 ± 14.04, with an onset of symptoms at 30.30 ± 10.65 years. The male-to-female ratio in Cluster 5 is 0.5:1. Remarkably, this cluster is characterized by a universal presence of both oral and genital ulcers (100%) and a noteworthy occurrence of ocular manifestations (38.9%). Additionally, neurologic involvement (16.7%) was higher in this cluster.

## Discussion

This study examined gender disparities in clinical manifestations and utilized cluster analysis to outline distinct clinical phenotypes in patients with BS. Our findings highlight a distinct clinical profile for male and female patients with BS. Specifically, male patients exhibited a higher likelihood of presenting with papulopustular lesions and ocular involvement, whereas female patients demonstrated a more frequent occurrence of genital ulcers. The identification of these gender-specific differences not only enhances our understanding of the heterogeneous clinical presentations of BS but also allows healthcare providers to develop individualized treatment approaches that address each patient’s unique needs. This personalized approach may also improve patient outcomes by ensuring that both genders receive care specifically suited to their clinical presentations.

Behçet’s symdrome predominantly occurs in populations with historical roots along the Silk Road [4]. Until now, differences in clinical manifestations between males and females with BS have been examined across different geographical regions, such as the Middle East, East Asia, and Europe [6, 11–15, 17]. In the Middle East region, one notable multicenter retrospective investigation in Egypt reported a higher prevalence of acne/pseudofolliculitis and vascular involvement in males. Conversely, central nervous system involvement, encompassing encephalitis and cranial nerve lesions, was more frequently observed in females [13]. Another study conducted in Iran, involving patients with BS, demonstrated that female BS had a higher prevalence of genital aphthosis, arthralgia, neurological manifestations, and cardiovascular disease in comparison to male patients [6]. Following these findings, the recent investigation of BS in Saudi Arabia revealed an increased prevalence of neurological manifestations in female BD patients, in contrast to papulopustular lesions being more prominent in male BS patients. Our findings align with previous research in the Middle East, particularly regarding the higher prevalence of pseudofolliculitis in male BS patients and genital aphthosis in females. However, it is noteworthy that in our study, there were no statistically significant differences in neurologic, vascular, and joint involvement in both genders.

In the East Asia region, an examination of newly diagnosed BS patients in Japan revealed a notable prevalence of ocular involvement in young adult males, contrasting with a significant occurrence of genital ulcers in females [14]. Furthermore, a comprehensive retrospective study conducted in China, underscored that male BD patients displayed a heightened likelihood of vascular involvement compared to female BD [15]. Another retrospective review focusing on BS in Korea demonstrated a higher frequency of ocular and vascular involvement in male BS patients, while skin manifestations, particularly erythema nodosum and neurologic involvement, were more commonly observed in females. Our findings align with existing research in East Asia, particularly regarding the higher prevalence of ocular involvement in male BS patients and genital ulcers in females. Nevertheless, other manifestations, including neurologic, vascular, and erythema nodosum findings, did not show significant gender disparities.

According to epidemiological data, BS displays a lower prevalence in the European continent, displaying a decreasing gradient from south to north, in contrast to higher rates observed in Turkey, Asia, and the Middle East region [18–21]. Bonitsis et al. investigated gender-related difference in clinical manifestations in patients with BS using a comprehensive German Adamantiades-Behçet’s Syndrome registry. Their findings revealed a clinically significant association, particularly in the higher risk of cardiovascular and ocular involvement among male patients with BD compared to female patients [12]. In a recent study focusing on gender-specific clinical variations within the Italian BS cohort, a higher incidence of papulopustular eruptions, posterior uveitis, and deep vein thrombosis was observed in males. Conversely, erythema nodosum-like lesions and arthralgia were more prevalent in females [17]. Upon comparing our results with these studies, we found that both ocular and papulopustular lesions were more frequently observed in male BS patients, with no significant differences in cardiovascular, vascular, erythema nodosum-like skin lesions, and joint involvement in both genders.

Until now, a limited number of studies have reported gender differences in BS across various regions of Turkey [22–26]. The main findings of our study suggested that the higher prevalence of genital ulcers in female BD and the increased occurrence of ocular involvement in male BD were consistent with some previous reports in Turkey [22, 24]. Additionally, joint involvement was reported with similar frequency in both genders [22], aligning with our study. However, unlike some other studies in Turkey [22–24, 26], we did not find a significant gender disparity in vascular or neurological involvement.

To the best of our knowledge, this is the first study to assess the cluster phenotype of patients with BS in the Eastern Black Sea Region of Turkey. In prior studies conducted in other region of Turkey, various clusters of adult patients with BS were identified, such as genital ulcers, erythema nodosum, papulopustular lesions and joint involvement, deep vein thrombosis and superficial vein thrombosis, musculoskeletal, uveitis, cerebral venous sinus thrombosis, peripheral major vessel disease, enthesopathy, acne, and arthritis [3, 27–32]. Beyond these reported clusters, two studies from East Asia highlighted the presence of neurological and gastrointestinal involvement types [33, 34]. In our current study, we demonstrated five clusters including cardiovascular type, ocular type, skin type, joint involvement type, and neurologic and mucosal involvement type. Cardiovascular type, ocular type, and skin type predominantly featured male patients, while joint involvement type and neurologic and mucosal involvement type were more prevalent among female patients with BS. The existence of distinct clusters of symptoms suggests that the development of BS may be influenced by multiple biological pathways.

Although the factors underlying the gender differences in clinical manifestations in BS remain unclear, several studies propose hypotheses suggesting that these differences may be attributed to a complex interplay of genetic, hormonal, immunological, environmental, and regional or ethnic factors. Genetic differences, particularly in the HLA region, have been reported, indicating a potentially significant role in the distinct clinical presentations observed between genders [10]. Additionally, hormonal factors are likely contributors to the gender-related clinical variations in BS, as reported by the slightly increased association of men with papulopustular lesions and women with genital ulcers [12, 35, 36]. Variances in environmental factors, such as exposure to infectious agents or dysbiosis in the intestinal flora, may play a role in modifying the clinical course and gender disparities in BS presentation [37–39]. Therefore, further studies are needed to assess the associated factors contributing to gender differences in clinical manifestations among patients with BS.

This study provides valuable insights into BS within the Eastern Black Sea Region of Turkey. Comparing our findings with studies from diverse geographical regions, especially those in the Middle East, East Asia, and Europe, contributes to a more comprehensive understanding of gender-related clinical variations in BS. The implementation of cluster analysis enhances the identification of distinct clinical phenotypes, offering a nuanced perspective on BS manifestations. However, the retrospective nature of our analysis introduces inherent limitations, primarily related to potential biases associated with data collection and missing information, which may impact the generalizability of our findings. Additionally, the observational nature of our study precludes the establishment of causality or temporal relationships between variables. Therefore, future research, including prospective studies with larger cohorts and longer follow-up periods, are warranted to validate our findings and elucidate the underlying mechanisms driving the observed clinical phenotypes in BS.

In conclusion, this study unveils diverse clinical features of BS between genders. Specifically, male patients exhibited a higher likelihood of presenting with papulopustular lesions and ocular involvement, whereas female patients demonstrated a more frequent occurrence of genital ulcers. Additionally, cardiovascular, ocular, and skin clinical phenotypes were predominantly featured in male patients. Understanding these gender-specific patterns is crucial for optimizing care, improving outcomes, and potentially tailoring treatment strategies in this complex disorder.

## Data Availability

Data are available on request due to privacy or other restrictions.
